# MicroRNA and Heart Failure: A Novel Promising Diagnostic and Therapeutic Tool

**DOI:** 10.3390/jcm13247560

**Published:** 2024-12-12

**Authors:** Andrea D’Amato, Silvia Prosperi, Paolo Severino, Vincenzo Myftari, Michele Correale, Pasquale Perrone Filardi, Roberto Badagliacca, Francesco Fedele, Carmine Dario Vizza, Alberto Palazzuoli

**Affiliations:** 1 Department of Clinical, Internal, Anesthesiology and Cardiovascular Sciences, ‘Sapienza’ University of Rome, Policlinico ‘Umberto I’ of Rome, 00161 Rome, Italy; andrea.damato@uniroma1.it (A.D.); silviapro@outlook.it (S.P.); paolo.severino@uniroma1.it (P.S.); vincenzo.myftari@gmail.com (V.M.); roberto.badagliacca@uniroma1.it (R.B.); dario.vizza@uniroma1.it (C.D.V.); 2 Cardiothoracic Department, ‘Policlinico Riuniti’ University Hospital, 71100 Foggia, Italy; 3 Department of Advanced Biomedical Sciences, Section of Cardiology, Federico II University, 80131 Naples, Italy; pasquale.perrone@unina.it; 4 IRCCS San Raffaele Cassino, 03043 Cassino, Italy; francesco.fedele@uniroma1.it; 5 Cardio Thoracic and Vascular Department, ‘S. Maria alle Scotte Hospital’, University of Siena, 53100 Siena, Italy; palazzuoli2@unisi.it

**Keywords:** heart failure, miRNAs, fibrosis, hypertrophy, biomarkers, diagnosis, therapy

## Abstract

Heart failure (HF) has a multifaceted and complex pathophysiology. Beyond neurohormonal, renin–angiotensin–aldosterone system, and adrenergic hyperactivation, a role for other pathophysiological determinants is emerging. Genetic and epigenetic factors are involved in this syndrome. In many maladaptive processes, the role of microRNAs (miRNAs) has been recently demonstrated. MiRNAs are small endogenous non-coding molecules of RNA involved in gene expression regulation, and they play a pivotal role in intercellular communication, being involved in different biological and pathophysiological processes. MiRNAs can modulate infarct area size, cardiomyocytes restoration, collagen deposition, and macrophage polarization. MiRNAs may be considered as specific biomarkers of hypertrophy and fibrosis. MiRNAs have been proposed as a therapeutical tool because their administration can contrast with myocardial pathophysiological remodeling leading to HF. Antimir and miRNA mimics are small oligonucleotides which may be administered in several manners and may be able to regulate the expression of specific and circulating miRNAs. Studies on animal models and on healthy humans demonstrate that these molecules are well tolerated and effective, opening the possibility of a therapeutic use of miRNAs in cases of HF. The application of miRNAs for diagnosis, prognostic stratification, and therapy fits in with the new concept of a personalized and tailored approach to HF.

## 1. Introduction

Heart failure (HF) is a complex syndrome characterized by a multifaceted pathophysiology [1,2]. Several pathways are involved in HF, such as neurohormonal activation, inflammation, renin–angiotensin–aldosterone system and adrenergic hyperreactivity [3]. Despite the recent advances in the knowledge of the pathophysiology and pharmacology of HF, it is associated with a high burden of mortality and morbidity [4]. Research on new biomarkers, for early diagnosis and prognostic stratification, as well as new therapeutic targets are required to improve the standard of care of HF patients [3]. In the last decades, attention has been focused on other mechanisms involved in HF, particularly its genetic and epigenetic determinants [5]. Among them, a role for microRNAs (miRNAs) has been pointed out [6]. They may have a role in predisposing patients to several pathophysiological processes, such as myocardial hypertrophy and fibrosis, leading to HF [6]. Consequently, they may represent a therapeutic target to avoid HF development and progression [6].

MiRNAs are small endogenous non-coding molecules of RNA involved in gene expression regulation, playing a pivotal role in intercellular communication [7,8]. Changes in miRNAs’ expression can cause the silencing of genes by inhibiting complementary messenger RNAs (mRNAs), involved in different biological processes, causing human disorders [7,9]. Many of the functions of miRNAs are still unknown and under study, opening the possibility of an innovative and personalized approach to disease.

The main origin of miRNA synthesis is the nucleus of the cell: transcription is allowed by the enzyme RNA polymerase II, then an initial maturation process takes place through the enzyme RNAse (Drosha), and the precursor (pri-mi-RNA) is exported through pores to the cytoplasm [7,10]. In the cell cytoplasm, the maturation of the miRNAs is completed [7,9]. It has been postulated that an ancient alternative synthesis route has been preserved during evolution that does not use the enzyme RNAse but uses introns that act as pri-mi-RNA and are called “mirtrons” [11].

The appeal of miRNAs lies in the fact that although they have complex functions they are “simple” molecules because they do not undergo post-processing modifications [11]. Once processed, miRNAs may be found in some organs [12,13] or may circulate in extracellular fluids as circulating miRNAs [12,14]. A small percentage of the latter is secreted as exosomes, macrovesicles that protect against degradation and allow miRNAs to enter other cells even at a distance [7,15,16]. Most of the form of the circulating miRNAs is bound to Argonaute 2 proteins (Ago2) [1,17], nucleophosmin 1 (NPM 1), and high-density lipoproteins (HDL) [7,18].

MiRNAs are tissue-specific and for this feature they are considered valid biomarkers [7,19]. For example, there is a specific miRNA isoform for the liver [7,20] or for the cartilage [7,21]. MiRNA levels are also generally stable and non-invasively assayable in biological fluids. Sensitive changes in their levels occur in case of disease, allowing for diagnosis and/or the evaluation of the effectiveness of therapy. For this reason, the most extensive current use of miRNAs is for oncological disease. In fact, miRNAs are considered very useful onco-markers and have helped the early diagnosis and therapy evaluation of many cancers [22].

Because of these characteristics, it is not difficult to hypothesize that these same miRNAs may also be useful and applicable for the diagnosis and therapy evaluation of different pathologies, such as heart diseases. Studying miRNAs, indeed, may offer profound insights into the intricate molecular landscape of cardiac pathologies, paving the way for precision medicine strategies and advancements in cardiovascular care.

The objective of this review is to highlight the current evidence of the application of miRNAs in cardiological diseases and especially in HF. Since HF is still the main cause of death in industrialized countries, every effort must be made to make diagnoses as early as possible and to find valid tools for treatment to significantly improve prognoses.

## 2. MicroRNAs as Potential Biomarkers in Heart Failure and Cardiovascular Diseases

The burden of cardiovascular disease is continuously increasing [23]. Despite advances in diagnosis and treatment, cardiovascular diseases represent one of the main causes of mortality and morbidity worldwide [23]. HF is a complex syndrome characterized by a multifaceted pathophysiology [24]. Beyond the traditional approach based on the identification of clinical signs and symptoms, as well as morpho-functional myocardial alteration, a deep knowledge of the genetic and epigenetic aspects of HF is required. The genetic characterization of different pathophysiological processes at the basis of HF may induce a reformulation of the HF concept, which is actually based on simplistic assumptions, such as its classification based on left ventricular ejection fraction (LVEF) or its symptoms [24,25,26]. In this regard, the role of miRNAs as diagnostic biomarkers, and prognostic and therapeutic tools, is potentially interesting in terms of improving the quality of care of HF patients in the future [26].

### 2.1. MicroRNAs and Cardiometabolic Aspects

Several miRNAs are involved in the regulation of metabolic processes in the myocardium [27,28,29,30,31,32,33,34,35,36,37,38,39,40,41,42,43,44,45,46,47,48].

MiR-22 is the most represented miRNA in the heart. It regulates calcium reuptake by sarcoplasmic reticulum, modulating sarcoplasmic/endoplasmic reticulum Ca^2+^-ATPase (SERCA2) activity [27,28]. It is markedly associated with hypertrophy and myocardial fibrosis [27,29], as it is implicated in the regulation of the transforming growth factor (TGF) pathway and in extracellular signal-regulated kinase (ERK) and mitogen-activated protein (MAP) kinase. Other important heart-related sights are miR-1, the regulator of calmodulin and smooth muscle cells; miR-208b, associated with structural titin [27,30]; and miR-181b, a marker of cardiomyocyte hypertrophy [31].

Many cardiovascular diseases are the result of dysfunction in heart metabolism. Several miRNAs are involved in the regulation of cardiac energy metabolism and are therefore potential therapeutic targets [27,28,29,30,31,32,33,34,35,36,37,38,39,40,41,42,43,44,45,46,47,48]. There is a relationship between glucose transporter 4 (GLUT4) and some miRNAs. MiR-133 and miR-223-3p, if silenced, decrease the expression of GLUT4 and increase the absorption of the glucose of the myocardium in patients with HF. MiR-200a-5p inhibits stress-associated selenoproteins, therefore it is associated with the dysregulation of glucose absorption in myocardial cells and eventually with cardiac hypertrophy [32,33]. In fact, cardiac hypertrophy is a complex process, partly still unknown, governed by miR-1, -133a, and -208 [31,34,35], and by insulin-like growth factor 1 (IGF-1) [34,36]. However, the precise role of these actors is still questionable. Hua et al. [34] found that IGF-1 deficiency mitigated cardiac hypertrophic remodeling, myocardial contractile change, and the overexpression of cardiomyocytic hypertrophy, induced by miR-1 and miR-133a. Another miRNA, and glucose transporters, was studied by Lopes et al. [32,37] who highlighted an upregulation of miR-17 expression in diabetic cardiac myopathy. In diabetic heart disease, cardiomyocytes undergo apoptosis via miR-34a which acts on the pro-survival protein sirtuin 1 (SIRT1) [32,38].

The abnormal expression of miRNAs has been associated with the presence and development of cardiometabolic diseases. Indeed, not only glucose metabolism, but also the β-oxidation of fatty acids, an important cardiac energy resource, is constantly regulated by miRNAs [32]. MiRNAs play an important role in facilitating the energetic adaptation of cardiomyocytes in the initial stage of ischemic damage and thus have an impact in determining the subsequent HF development [39,40]. All pathological stimuli, not only ischemia, can cause stress and hypoxia and force cardiomyocytes to enact a series of adaptive responses that in the long run become dysfunctional [39,41,42]. In cases of stress, it is known that a metabolic shift occurs from fatty acid oxidation (FAO) to glycolysis [39,40,43]. Many HF models are associated with low FAO levels and increased glycolysis levels [39,41,44,45]. MiRNAs perform an important regulatory action in this “energy shift”, a fundamental point in the genesis and development of HF. The hypertrophic heart is more dependent on glucose because it is a more efficient source of energy [46,47] and this metabolic shift has been suggested as a beneficial therapy [46,48].

### 2.2. MiRNAs in Myocardial Inflammation and Ischemia

Many miRNAs may play a role in myocardial inflammatory and ischemic damage, demonstrating a pivotal role in the pathophysiological processes leading to HF.

**Figure 1:**
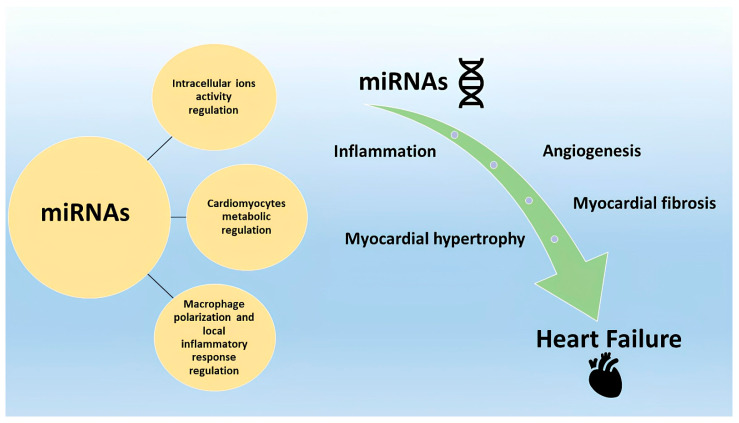
**Molecular and pathophysiological mechanisms in which microRNAs (miRNAs) are involved in the myocardium.** MiRNAs are involved in several molecular processes in the myocardium, such as ion activity, and metabolic and inflammatory response regulation. MiRNAs contribute to heart failure through four main processes in the myocardium: inflammation, angiogenesis, myocardial hypertrophy, and fibrosis. MiRNAs: microRNAs.

The application of miRNAs has gained interest in the field of HF treatment, particularly regarding cases of HF with preserved ejection fraction (HFpEF). The complexity of pathophysiology and the lack of specific therapy for HFpEF made it necessary to seek insight into a genetic and molecular basis for predicting its pathophysiology and clinical evolution [49]. Hahn et al. [50] identified, by endomyocardial biopsy, a specific transcriptome in HFpEF patients, which differentiates them from healthy controls and HF patients with reduced ejection fraction (HFrEF) [50]. In HFpEF patients, an upregulation of energy-producing genes was found. On the contrary, other genes involved in angiogenesis, autophagy, and endoplasmic reticulum activity were downregulated. Based on transcriptomes, two subgroups of different HFpEF entities were identified: one comprising female patients with an upregulation of gene pathways related to inflammation and myocardial hypertrophy, the other with molecular characteristics comparable to patients with HFrEF [50]. In HFpEF, there is an increased expression of pro-fibrotic markers, such as galectin-3, the soluble type of interleukin-1 (IL-1) receptor, and suppression of tumorigenicity 2 (ST2), as well as a reduced expression of enzymes involved in extracellular matrix destruction, such as metalloproteinase-2 [51,52]. Several miRNAs are specifically expressed in HFpEF patients: hsa-miR-181a-2-3p, miR-3908, miR-3135b, hsa-miR-30a-5p, hsa-miR-199b-5p, hsa-miR-5p-106a-5p, hsa-miR-486-5p, hsa-miR-191-5p, hsa-miR-193a-5p, and hsa-miR-660-5p [51,52]. MiR-101 regulates fibrogenesis, acting on the TGF pathway [51,52], while miR-146a regulates the nuclear factor kappa B (NF-κB) pathway, inflammation, and subsequent myocardial fibrosis [53]. Macrophage inflammatory cells play an important role: HFpEF is characterized by systemic inflammation, during which macrophages change their state of polarization and induce a reverse cardiac remodeling in the subsequent phase [27,53]. The polarization of M2 macrophages is associated with immunosuppression, fibrosis, and tissue repair, while M1 is associated with inflammation and cell death [54]. Several miRNAs regulate the two distinct macrophage polarities: miR-9, miR-125b, miR-127, and miR-155 are involved in macrophage M1 polarization, while miR-34a, miR-125a-5p, miR-124, miR-132, miR-223, and miR-146a are involved in the M2 polarization of the myocardial macrophages [27,53]. The main molecular and pathophysiological mechanisms in which miRNAs are involved in the myocardium are represented in Figure 1.

Song et al. [55] demonstrated a protective role against ischemia–reperfusion injury for miR-210 which may prevent cardiac dysfunction after myocardial infarction. They found that, in cases of myocardial ischemia and reperfusion, there is a deficiency of miR-210 which promotes oxygen consumption by mitochondria with consequent reactive oxygen species (ROS) production. In this condition, a reduction of adenosine triphosphate (ATP) production and an increase in proton leaks have been observed. The increase in proton leaks is associated with mitochondrial damage and cell death. Mitochondrial and energetic dysfunction is one of the main molecular mechanisms involved in HFrEF.

Li et al. [56] reported that also miR-20b-5p is involved in ischemia–reperfusion damage, after myocardial infarction. In particular, the antioxidant resveratrol enhances mitochondrial function and protection from ischemia–reperfusion damage, through the inhibition of the stromal interaction molecule 2 and consequent improved expression of miR-20b-5p.

Several miRNAs have been associated with disease evolution and prognosis in myocarditis and inflammatory cardiomyopathy [57,58,59,60,61,62,63]. Chimenti et al. [57] focused on the role of miRNAs in myocarditis. They identified intracellular miRNAs, observed in myocardial biopsies, and extracellular miRNAs, identified in blood samples. Regarding intracellular miRNAs, miR-1, miR-133a, and miR-133b are involved in cardiomyopathy and myocarditis. In particular, miR-133a is associated with myocardial recovery and a reduced risk of arrythmias and HF development in inflammatory cardiomyopathy [57,58]. MiR-208a is upregulated in the acute phase of myocarditis, while the levels of MiR-208b during the sub-acute phase of myocarditis have been related to left ventricular function recovery [57,59].

During myocarditis, several miRNAs are involved in viral replication and in the response of the host immune system to viral infection, leading to HF. MiR-221 and miR-222 deficiency promotes cardiac damage induced by inflammation [57,60], miR-203 stimulates cardiomyocyte apoptosis through the nuclear factor interleukin-3 [57,61], and miR-21 and miR-125b are involved in myocardial fibrosis. [57,62]

Circulating extracellular miRNAs have been associated with myocarditis severity and prognosis. In this regard, miR-208b and miR-499-5p have been associated with troponin T values in patients with cytomegalovirus-related myocarditis [57,63].

In summary, to understand the close link between miRNAs and the cardiovascular system, it has to be considered that the suppression of all miRNAs can lead to an early death and to the development of severe heart diseases [46,64]. Research on genetic and epigenetic alterations is a key point for building a customized approach to the diagnosis and management of HF in general.

## 3. MicroRNAs as Potential Therapeutic Targets in Heart Failure

MiRNAs are involved in several pathophysiological processes leading to HF, such as myocardial hypertrophy, myocardial fibrosis, inflammation, and angiogenesis [65] (Figure 1). MiRNAs are crucial in the balance between cardiomyocytes and extracellular matrix. Their over or under-expression in failing hearts leads to a vicious cycle which promotes and maintains the structural and functional myocardium alterations of HF [65,66]. This has been observed both in chronic HF, and also in acute HF, following, for example, a myocardial infarction. In this context, several miRNAs are involved in the imbalance of many signaling pathways contributing to the early myocardial remodeling after an ischemic event. This occurs through the regulation of the infarct area size, the cardiomyocytes restoration, the collagen deposition, and the macrophage polarization [67]. The ultrastructural modifications are associated with hemodynamic and clinical effects, influencing diastolic and systolic function, and myocardial contractility [68]. For this reason, miRNAs may represent a therapeutic target to revert these processes, combatting HF.

Currently, no miRNAs have been approved for clinical use. However, they are a potential therapeutic option for several reasons. They are endogenous molecules regulating several physiological and molecular processes; targeting mRNAs, they can be used to enhance or inhibit precise pathophysiological pathways involved in many diseases; the complexity of human miRNome is low, and, therefore, the development of miRNA mimics and antimirs as therapeutic weapons is feasible [69]. In cardiology, however, several small interfering RNAs (siRNAs) have been approved for the treatment of many diseases. SiRNAs are silencing/interfering molecules comparable to endogenous miRNAs, able to enhance the activity of the RNA interference pathways leading to the downregulation of a specific pathophysiological pathway [69]. In clinical practice, Patisiran has been approved for the treatment of hereditary transthyretin amyloidosis and Inclisiran has been approved for the treatment of hypercholesterolemia [69].

How can levels of tissue and circulating miRNAs be regulated? And how can miRNAs be delivered to the target organ? MiRNA mimics are synthetic double-strand oligonucleotides which may be administered in association with a vector, such as an Adenovirus. They are converted to a single-strand miRNA binding the mRNA 3′ untranslated region. MiRNA mimics restore poorly expressed miRNAs [65]. Antimirs represent a single-strand antisense oligonucleotide. They may contrast miRNAs’ function, inhibiting the bind between a miRNA and the mRNA target. Antimirs may be administered subcutaneously and intravenously, and they remain stable, also in terms of intracellular behavior, taking their effect in several days [65].

The use of miRNA mimics and antimirs has been hypothesized as a therapeutic approach to regulate the imbalanced miRNA expression in HF. Although several mimics and antimirs have been used with success in animal models, some important gaps have to be filled to use them as a therapy in humans [43]. For example, their pharmacokinetic and pharmacodynamic features are not fully understood, nor are possible side effects. The administration of mimics and antimirs may produce side effects in non-target organs, making their administration more dangerous than beneficial [43]. To overcome this problem, it is necessary to deliver the drug to the target organ. In animal models, the delivery of mimics and antimirs to the target organ is feasible through different mechanisms, such as using macrovesicles and exosomes, or through catheter-based local delivery [43].

### 3.1. The Role of miRNAs as Potential Therapeutic Targets in Myocardial Fibrosis

**Figure 2:**
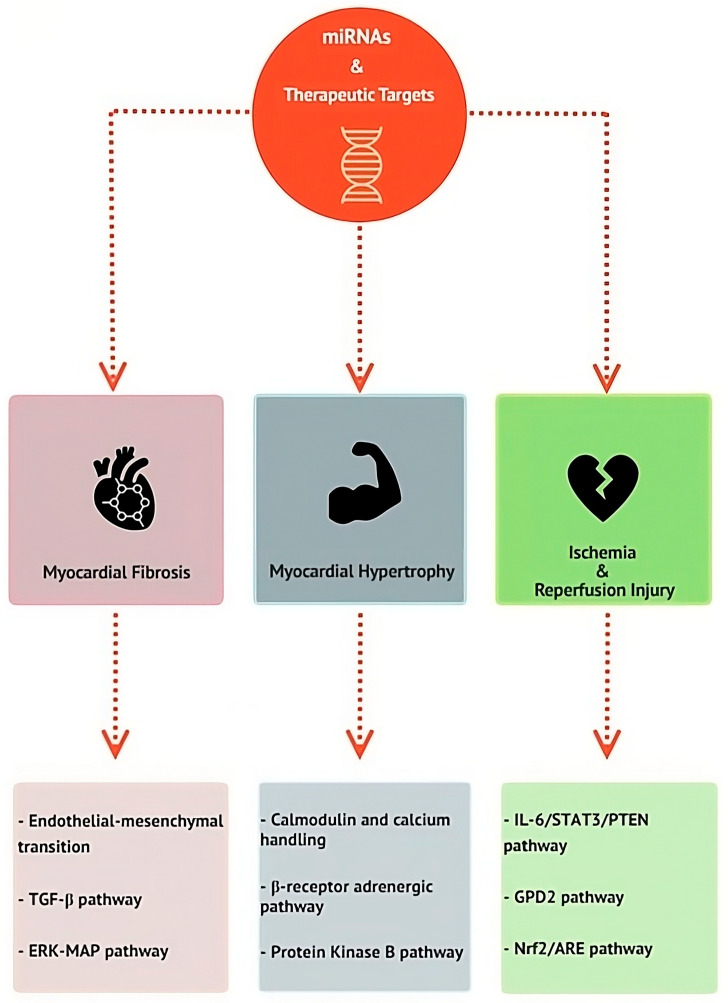
**Main pathophysiological mechanisms on which microRNAs (miRNAs)-targeted therapy has been studied.** Myocardial fibrosis, hypertrophy, and ischemia are the main mechanisms leading to heart failure and, for this reason, they may represent crucial mechanisms to counteract. Many molecular pathways have been involved in each mechanism. MiRNAs: microRNAs; TGF-β: transforming growth factor- β; ERK-MAP: extracellular signal-regulated kinase/microtubule-associated protein kinase; IL-6/STAT3/PTEN: interleukin-6/signal transducer and activator of transcription 3/Phosphatase and tensin homolog; GPD2: Glycerol-3-Phosphate Dehydrogenase 2; Nrf2/ARE: nuclear erythroid 2-related factor 2.

Fibrosis is a myocardial remodeling process determined by collagen deposition with a cardiomyocyte’s substitution common to several pathological conditions predisposing to HF [70]. The fibrosis distribution differs according to the considered condition. Myocardial infarction is characterized by localized fibrosis, while myocarditis, hypertensive cardiopathy, and genetic cardiomyopathies are characterized by diffuse fibrosis [70]. Several miRNAs have been involved in promoting the fibrosis process, thus representing potential therapeutic targets. MiR-21 family overexpression has been observed in aortic stenosis and myocardium stress [71], and it leads to endothelial–mesenchymal cell transition induced by transforming growth factor-β (TGF-β) [72] (Figure 2). MiR-21 is also involved in HF-related fibrosis through the stimulation of the ERK-MAP pathway (Figure 2). Silencing miR-21 through an antimir was associated with reduced ERK-MAP activity, with reduced fibrosis contrasting the myocardial dysfunction [73]. MiR-21 is largely expressed in human cells, and it is one of the most studied miRNAs. Bejerano et al. [74] delivered miR-21 through nanoparticles in macrophages inside the infarct area. MiR-21 induced a transition in macrophage phenotype, from pro-inflammatory to reparative, stimulating angiogenesis and reducing fibrosis and myocardial remodeling. MiR-24 is downregulated after a myocardial infarction, and it is associated with fibrotic remodeling and apoptosis. The integration of miR-24 in animal models is associated with a reduced necrotic area size and improved cardiac function [75].

### 3.2. The Role of miRNAs as Potential Therapeutic Targets in Myocardial Hypertrophy

Myocardial hypertrophy is a maladaptive and reactive process to several pathological stimuli. It may be due to a genetic mutation, as often occurs in sarcomeric gene variants in hypertrophic cardiomyopathy, or due to conditions of pressure overload, as occurs in aortic stenosis and hypertensive cardiomyopathy. From a pathophysiological point of view, myocardial hypertrophy is associated with diastolic dysfunction. Given its role in myocardial disease, the possibility to target pro-hypertrophic molecular processes is a main investigation focus.

MiR-1 is strongly involved in regulating myocardial hypertrophy. Through the regulation of calmodulin and calcium-handling genes, miR-1 overexpression may reverse hypertrophy (Figure 2). In this regard, the administration of miR-1 mimics induces a reverse hypertrophic remodeling in experimental models [76]. MiR-133 is a regulator of myocardial hypertrophy and of the β receptor adrenergic pathway (Figure 2). It is one of the main miRNAs expressed in the heart and its expression is closely related to myocardial infarction. It acts upstream in numerous signaling pathways, and, for this reason, its role is controversial [67]. A stable level of miR-133 counters myocardial hypertrophy through the activation of the protein kinase B pathway [35] (Figure 2). It also reduces myocardial fibrosis and improves cardiac diastolic function [77]. However, miR-133 is crucial in maintaining the balance of cardiac function because it is involved in several molecular processes. It may represent a therapeutic target because it may promote therapeutic myocardial remodeling, inhibiting fibrosis, inflammation, and compensatory hypertrophy in ischemic hearts [67]. Also, the miR-212/132 family is associated with cardiac hypertrophy and HF. The administration of miR-132 antimirs reduced myocardial hypertrophy and the HF development [78].

### 3.3. MiRNAs as Therapeutic Targets in Myocardial Ischemia and Ischemia Reperfusion Injury

Studies involving miRNAs as therapeutic targets have been reported in myocardial ischemia, ischemia–reperfusion injury, and HF [68,79,80,81,82,83,84,85,86,87,88].

Batkai et al. [68] demonstrated the feasibility and therapeutic efficacy of the intravenous administration of an oligonucleotide inhibitor of miR-132, named CDR132L, in an animal model of HF post-acute myocardial infarction. The treatment with CDR132L led to reverse myocardial remodeling, reduced fibrosis, and maladaptive compensatory hypertrophy. All these aspects determined clinically relevant effects. In particular, an improvement in LVEF, assessed by cardiac magnetic resonance, and an improvement in myocardial contractility and systolic function, assessed invasively by a pressure–volume loop, have been observed [68]. Taubel et al. [79] conducted the first in-human phase 1b trial using a synthetic oligonucleotide inhibitor of miR-132 (CDR132L), demonstrating that it was safe, and that it reduced the levels of N-terminal pro B-type natriuretic peptide (NT-proBNP) and fibrosis markers, and induced QRS narrowing. Antimir-132 administration was associated with an improvement in LVEF and end systolic volume in experimental models of HF following myocardial infarction [80,81,82].

MiR-320 mimic use may promote interstitial fibrosis in mice models of transverse aortic constriction [81]. In this regard, miR-320 is involved in many pathophysiological processes leading to HF. It promotes the interleukin-6/signal transducer and the activator of transcription 3/Phosphatase and the tensin homolog (IL6/STAT3/PTEN) pathway as well as cardiomyocyte lipotoxicity, stimulating fatty acid uptake [81,82,83] (Figure 2). The most interesting aspect is that miR-320 may have a different role according to the type of cell considered. MiR-320 overexpression in cardiac fibroblasts hampers the processes of fibrosis and hypertrophy. A lower expression of miR-320 in cardiomyocytes is associated with reduced cardiac function [82,84].

MiR-92a is upregulated in animal models after the induction of myocardial infarction. The intravenous administration of miR-92a antimir is associated with a smaller infarct size area and improved diastolic and systolic function [85]. Bellera et al. [86] demonstrated that the intracoronary infusion of miR-92a antimir has an anti-remodeling effect, reducing regional wall kinetic abnormalities following an acute myocardial infarction. In humans, the first study using MRG-110, an LNA-92a-3p inhibitor, included healthy subjects. MRG-110 reduced the miR-92 circulating level efficiently. Jin et al. [87] found that an miR-19a/19b infusion in animal models of post-myocardial infarction HF was associated with cardiomyocytes’ regeneration, the reduction of the infarct area, and improvement in cardiac function.

MitoQ is an antioxidant which specifically targets mitochondria. Song et al. [55] demonstrated that the administration of MitoQ in mice with miR-210 deficiencies reduced the risk of ischemia–reperfusion injury and cardiac dysfunction after acute myocardial ischemia. MitoQ improved mitochondrial energy, reducing the impact of miR-210 deficiency on ischemia–reperfusion injury after myocardial ischemia. They also identified Glycerol-3-Phosphate Dehydrogenase 2 (GPD2), a mitochondrial membrane-bound protein, as a miR-210 target (Figure 2). The deficiency of miR-210 determines the upregulation of GPD2 which is directly involved in mitochondrial ROS production and left ventricular dysfunction following ischemia–reperfusion injury [55,88].

Despite the potential role of a therapy based on endogenous miRNA regulation, several clinical trials involving miRNAs have been suspended and no miRNAs have been reported in a phase III clinical trial, due to side effects [89]. The latter are mainly caused by several features of miRNAs. In particular, their instability may lead to changes in molecular structure and a change in targets leading to inflammation system hyperactivation or coagulative disorders. Their negative charge may determine a long storage in human tissue with a reduced clearance. In the case of many miRNA targets, an off-target effect may be observed leading to unpredictable consequences [43,90]. The main miRNAs and their role in cardiac function and cardiac diseases are summarized in Table 1.

## 4. Current Challenges

The therapeutic application of miRNAs in cases of HF is hindered by several critical challenges, including immune system activation, limited delivery specificity, and off-target effects.

MiRNAs, when delivered as therapeutic agents, can trigger innate immune responses due to their similarity to viral RNA, especially toll-like receptors (TLR) [91]. This activation often leads to the production of pro-inflammatory cytokines, which can result in unwanted side effects or the immune rejection of the treatment [92]. Approaches that aim to reduce immune system activation, while maintaining therapeutic efficacy, are needed. They may include the modification of the nucleobases’ structure or the development of safe delivery system methods.

In fact, successfully delivering miRNAs to their targets and generating effective therapies relies on the crucial step of miRNAs crossing the myocardial cell membrane. This process is essential for ensuring that the miRNAs can reach their intended sites of action and exert their therapeutic effects [93]. Viruses, like lentiviruses, may be used to effectively deliver miRNAs to the myocardial cells. Yang et al. [94] demonstrated that the lentiviral delivery of miR-322 inhibition could reduce hypoxia-induced cardiomyocyte apoptosis. Although lentiviral-based miRNA therapy shows promising results for heart disease treatment, its potential for causing insertional mutagenesis limits its broader application. Inorganic nanoparticles, on the other hand, are considered strong candidates for efficient RNA delivery, even though there is still a lack of extensive research focused on the application of these vehicles in the cardiovascular setting [95].

## 5. Future Perspectives

Cardiovascular diseases are the main cause of death worldwide. In particular, HF is the terminal point of all cardiovascular diseases. The identification of new and more accurate biomarkers for diagnosis and prognosis in HF is one of the most important goals of current scientific research. In this regard, miRNAs may represent an interesting tool both for diagnostic and therapeutic implications. According to the pleiotropic role and multifaceted use of miRNAs, they represent a “theranostic tool” because they may assume both a therapeutic and diagnostic role in cardiovascular diseases [96]. Indeed, the possibility to use them in the regulation of gene expression and molecular processes allows their use in the following settings: (i) the early diagnosis of cardiovascular disease; (ii) the in-depth comprehension of pathophysiological processes occurring in complex conditions (i.e., HFpEF); (iii) disease prognosis (i.e., left ventricular function recovery and tachyarrhythmia risk in myocardial inflammatory disease); (iv) and the possible regulation of gene expression finalized to regenerative processes (i.e., myocardial infarction, HF). Two practical examples of particular interest for future application in clinical practice are the use of anti-miR-132 in HF and the inhibition of miR-92a in ischemia [85]. Anti-miR-132 may be important to face the adverse myocardial remodeling process that occurs in chronic HF, determining improvement in myocardial contractility, systolic function, and myocardial wall stress reduction [79,85]. The inhibition of miR-92a is associated with capillary and arterioles regeneration, improved perfusion, and a reduction in necrosis [85,86]. Despite the interesting rationale behind miRNAs’ use in cardiology, more evidence and studies are needed to define miRNAs as practical diagnostic and therapeutic tools. While miRNAs target multiple mRNAs, regulating the expression of several proteins, siRNAs—which are small molecules able to silence post-transcriptional genes—target specific mRNAs [97,98]. SiRNAs have indeed been approved for human use and their introduction in clinical practice has completely changed the treatment of many diseases, such as hypercholesterolemia, transthyretin amyloidosis, hepatic porphyria, and hyperoxaluria [97,98]. Regarding siRNAs, studies for many other molecules and for other diseases are currently ongoing [97,98].

## 6. Conclusions

MiRNAs are implicated in a variety of different pathologies, including HF. Some miRNAs are specifically represented in the heart and their levels reflect the presence or absence of some morbid conditions. The different expressions of tissue and circulating miRNAs are associated with several pathophysiological processes, such as hypertrophy and fibrosis, which may lead to HF [65]. In particular, pathophysiology and cardiopulmonary interaction in HFpEF are the main subjects of study [99], and several miRNAs may be considered distinctive pathophysiological markers of HFpEF [24]. Other miRNAs are involved in the myocardial remodeling processes following a myocardial infarction [65]. In particular, they regulate the infarct area and post-ischemic inflammation.

Understanding the role of miRNAs in HF and cardiac diseases is crucial for unraveling intricate molecular mechanisms, identifying therapeutic targets, and developing innovative diagnostic biomarkers. The potential to regulate circulating and tissue miRNAs through antimirs and miRNA mimics opens up the possibility of a therapeutic role for these small molecules. For this reason, miRNAs may represent a promising diagnostic, prognostic, and therapeutic tool for many cardiovascular diseases, particularly HF.

Their use as biomarkers and as a therapeutic tool in cardiology strengthens the importance of the concept of a personalized and patient-tailored approach to HF, mainly based on pathophysiological background [100]. However, more studies involving human subjects and investigating clinical aspects are required to achieve this goal.

## Figures and Tables

**Table 1:** Summary of the main microRNAs (miRNAs) and their role in cardiac function and cardiac diseases.

miRNA	Role in Cardiac Function and Pathology	Reference
miR-22	It modulates SERCA2 activity, the TGF pathway, and regulates myocardial hypertrophy and fibrosis	[27,28,29]
miR-181b	It is a marker of cardiomyocyte hypertrophy	[31]
miR-223-3p	It decreases the glucose absorption of cardiomyocytes	[32,33]
miR-200a-5p	It inhibits stress-associated selenoproteins, associated with cardiomyocyte hypertrophy	[32,33]
miR-17	It is upregulated in diabetic cardiomyopathy	[38]
miR-101	It regulates fibrogenesis regulation through the TGF pathway	[51,52]
miR-146a	It is involved in the NF-κB pathway, inflammation, and myocardial fibrosis regulation	[53]
miR-210	It may prevent cardiac dysfunction after myocardial infarction; its deficiency promotes mitochondrial oxygen consumption and ROS production	[55,88]
miR-20b-5p	It reduces ischemia–reperfusion damage; its expression is improved by resveratrol	[56]
miR-133a	It is associated with myocardial recovery, and reduced risk of arrhythmias and HF development in inflammatory cardiomyopathies	[57,58]
miR-208a	It is upregulated during the acute phases of myocarditis	[57,59]
miR-208b	It is related to left ventricular function recovery during the subacute phases of myocarditis	[57,59]
miR-221	Its deficiency promotes cardiac damage induced by inflammation	[57,60]
miR-222	Its deficiency promotes cardiac damage induced by inflammation	[57,60]
miR-203	It stimulates cardiomyocyte apoptosis through NFIL3	[57,61]
miR-21 and miR-125b	They are involved in myocardial fibrosis	[57,62]
miR-208b and miR-499-5p	Circulating miRNAs associated with myocarditis severity and prognosis	[57,63]
mirR-1	Its overexpression may reverse cardiac hypertrophy through calmodulin and calcium-handling genes	[76]
miR-132a	The use of anti-miR-132a is associated with an anti-remodeling effect, an improvement in myocardial contractility and systolic function, and myocardial wall stress reduction	[79]
miR-320	Its low levels are associated with reduced cardiac function	[84]
miR-92a	Its low levels are associated with left ventricular dysfunction and ischemia–reperfusion injury	[86]

SERCA2: sarcoplasmic/endoplasmic reticulum Ca^2+^-ATPase; TGF: transforming growth factor; NF-κB: nuclear factor kappa B; ROS: reactive oxygen species; NFIL3: nuclear factor, interleukin 3 regulated.
